# Methionine enkephalin(MENK) upregulated memory T cells in anti-influenza response

**DOI:** 10.1186/s12865-023-00573-0

**Published:** 2023-10-12

**Authors:** Jing Tian, Wenrui Fu, Zifeng Xie, Xiaonan Wang, Miao Miao, Fengping Shan, Xiaodong Yu

**Affiliations:** 1 Department of Immunology, School of Basic Medical Science, Jinzhou Medical University, Jinzhou, 121001 China; 2 Department of Immunology, School of Basic Medical Science, China Medical University, Shenyang, 110122 China; 3 Biostax Inc., 1317 Edgewater Dr., Ste 4882, Orlando, FL 32804 USA; 4 Department of Nursing, The First Affiliated Hospital of Jinzhou Medical University, Jinzhou, 121001 China

**Keywords:** Methionine enkephalin, Influenza A virus, Opioid receptor, Antiviral efficacy, Memory T cell

## Abstract

Novel prophylactic drugs and vaccination strategies for protection against influenza virus should induce specific effector T-cell immune responses in pulmonary airways and peripheral lymphoid organs. Designing approaches that promote T-cell-mediated responses and memory T-cell differentiation would strengthen host resistance to respiratory infectious diseases. The results of this study showed that pulmonary delivery of MENK via intranasal administration reduced viral titres, upregulated opioid receptor MOR and DOR, increased the proportions of T-cell subsets including CD8^+^ T cells, CD8^+^ T_EM_ cells, NP/PA-effector CD8^+^ T_EM_ cells in bronchoalveolar lavage fluid and lungs, and CD4^+^/CD8^+^ T_CM_ cells in lymph nodes to protect mice against influenza viral challenge. Furthermore, we demonstrated that, on the 10th day of infection, the proportions of CD4^+^ T_M_ and CD8^+^ T_M_ cells were significantly increased, which meant that a stable T_CM_ and T_EM_ lineage was established in the early stage of influenza infection. Collectively, our data suggested that MENK administered intranasally, similar to the route of natural infection by influenza A virus, could exert antiviral activity through upregulating T-cell-mediated adaptive immune responses against influenza virus.

## Introduction

Annual influenza vaccinations are considered to be effective to prevent influenza infection, which are mainly focused on inducing a strain-matched humoral immune response but require updates [1–3]. Antigenic drift can occur in all subtypes of influenza A virus, leading to the immune protection conferred by the host’s acquired immunity obtained through previous infections or immunization no longer being effective against the new variants; moreover, the recognition between antigen and antibody is no longer fully functional [4, 5].

In contrast, adaptive cellular immunity, especially cytotoxic T lymphocytes, targeted relatively highly conserved internal viral proteins such as the influenza nucleoprotein (NP), and overcomed the drawbacks of current antibody-inducing vaccines and could provide cross-protective immunity against emerging pandemic influenza viruses [6, 7]. Different studies have indicated the importance of memory T cells in preventing influenza reinfection [8–10], the number of circulating influenza specific memory CD8^+^ T cells is inversely correlated with viral load [11]. In addition, influenza viruses evolved resistance to antiviral drugs via multiple pathways [12]. A single amino acid S31N substitution in influenza M2 sequences lead resistant to the adamantane class of antivirals [13]. H274Y substitution of H1N1 viruses conferred resistance to oseltamivir, the drug resistant viruses rapidly disseminated globally and was particularly alarming [14]. And thus, there is an urgent need for development of prophylactic and therapeutic agents not affected by viral adaptation or mutation, which would supplement the antiviral drugs and vaccines.

In 1979, Joseph Wybran reported that methionine enkephalin (MENK) had different effects on human T cells because of the existence of different opioid receptor subtypes on these cells [15]. MENK as an immunomodulator of opioid peptides exerts pleiotropic effects on immune cell function, modifying immune responses to extracellular stimuli such as antigens, antibodies, and mitogens that cross-link the T-cell receptor [16]. Early studies reported that MENK could enhance B-cell and T-cell proliferation [17, 18]. Our team has demonstrated that MENK treatment upregulated the proportion of CD8^+^ T cells, prompted the expression of markers of T-cell activation, enhanced cytotoxic activity, and induced the production of IFN-γ [19] via the precise regulation of subunits of opioid receptors MOR and DOR [20]. MENK inhibited the activity of Tregs and retarded tumor growth by downregulating Tregs [21], while also regulating lymphocyte subsets in the peripheral blood by inhibiting Tregs [22]. However, to date, the immune function of MENK on memory T cells has not been evaluated.

Our previous study confirmed that MENK exerted anti-influenza virus activity by regulating innate immunity in vivo and in vitro [23, 24]. In light of previous research findings, we hypothesize that adaptive immunity, especially cytotoxic T cells, plays indispensable role in MENK’s protective mechanism against influenza infection. Therefore, the focus of this study was to elucidate the cellular immune functions by which MENK acts and provide experimental support for MENK as an immune modulator for anti-influenza.

## Materials and methods

### Mice and virus

Female C57BL/6 mice (6–8 weeks old) were were obtained from the Laboratory Animal Center of Jinzhou Medical University. All animal experiments were carried out in accordance with the guidelines of the Animal Use and Care Committee of Jinzhou Medical University. The mouse-adapted influenza strain A/PR/8/34 (H1N1; PR8) was obtained from the China Center for Disease Control and Prevention (Beijing, China). The virus was propagated in 10-day-old embryonated hens’ eggs as described previously [25].

### Reagents

MENK (≥ 99% purity) was provided by American Peptide Co. (Sunnyvale, CA, USA). RNeasy Mini Kit (74104) was purchased from Qiagen. One Step SYBR® Prime Script™ RT-PCR Kit (RRO66A) was purchased from TaKaRa. The mAbs of influenza A virus nucleoprotein (ab20343) were purchased from Abcam. DyLight®488 IgG (H + L) were purchased from Earthox. The peptides of influenza A NP366-374 (ASNENMETM) and PA224-233 (SSLENFRAYV) were purchased from AnaSpec, Inc. (Fremont, CA, USA). The mAbs used for flow cytometry were purchased from BD Biosciences. Fixation/permeabilization solution (554714) was purchased from BD Biosciences.

### Infection and treatment of mice

**Fig. 1:**
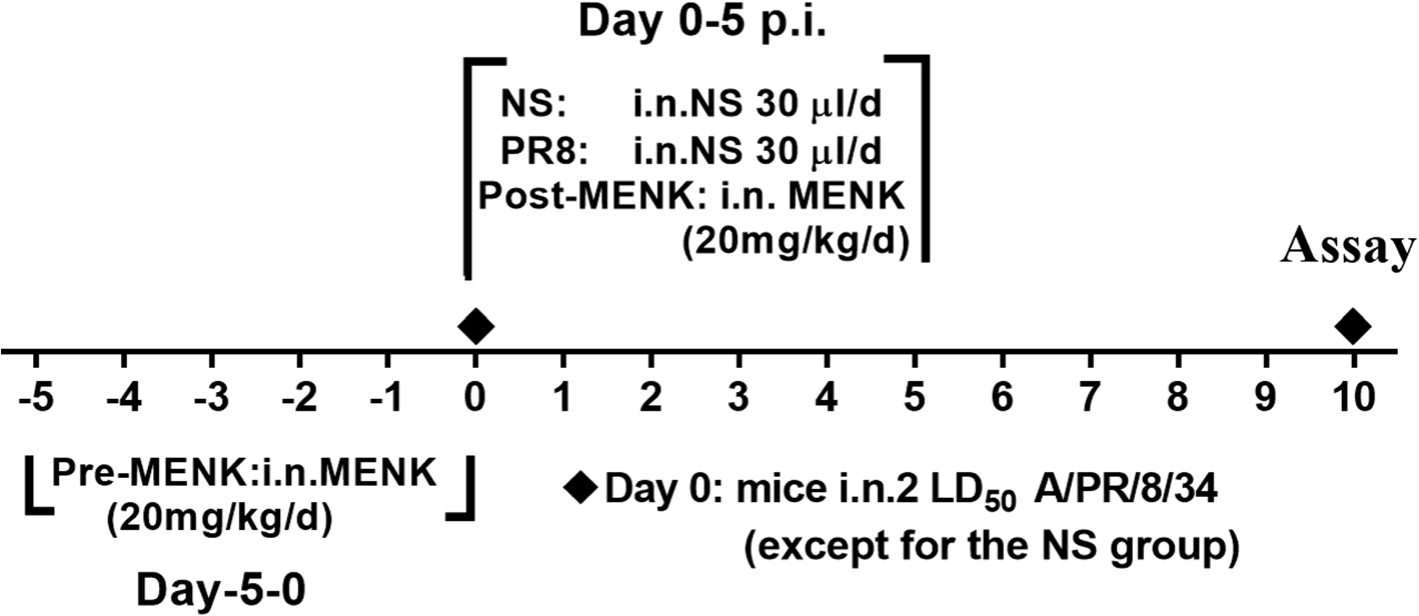
Experimental design. C57BL/6 mice were infected via intranasal instillation (i.n.) with 2 LD_50_ of influenza A virus (PR8) (d0), except for mice in the NS group. The mice in the pre-MENK group were intranasally administered 20 mg/kg MENK daily for 6 successive days (d-5 to d0) prior to infection, then infected with PR8 (d0). The mice in the post-MENK group were infected with PR8 (d0), and followed with 20 mg/kg MENK daily post-infection (d0 to d5 p.i.). The mice in the normal control group and PR8 group were treated with an equal volume of saline for 6 successive days (d0 to d5)

Female C57BL/6 mice were divided into four groups: normal saline (NS) group, influenza virus (PR8) model control group, pre-MENK group, and post-MENK group. Figure 1 provides an outline of the construction of the mouse model. The mice were anesthetized by isoflurane inhalation before inhaling of PR8 or MENK. The mice were placed in the induction box of the anesthesia machine and isoflurane was maintained at a concentration of 1–1.5%. After the mice were completely anesthetized, intranasal instillation were administered. On the 10th day of PR8 infection, the mice were euthanized for cervical dislocation and used for T cells experiments.

### Hemagglutinin (HA) test

On day 4 p.i., Lung tissue was homogenized to a 10% (w/v) suspension with sterilized PBS, and centrifuged at 12000 rpm for 10 min. The supernatant was serially diluted two fold with PBS, 50μL added to each U-bottom well, 50 μL of 1% guinea pig red blood cells, then mixed and incubated at room temperature for 30 min. The final dilution that completely agglutinated red blood cells was considered the end-point of titration.

### Immunofluorescence

On day 4 p.i., the left lung lobes of the mice in each group were fixed in 4% paraformaldehyde and then dehydrated, embedded in paraffin, and cut into 4-μm-thick sections for immunofluorescence staining. The right lobes were stored in liquid nitrogen for a virus titer test (HA) and qPCR analysis.

The sections were deparaffinized, rehydrated, and then subjected to antigen retrieval using EDTA at 94 °C for 20 min, supplemented with 3% hydrogen peroxide for 10 min, permeabilized with 0.5% Triton X-100, incubated with goat serum at 37 °C for 30 min, and then incubated with influenza NP at 1/100 dilution at 4 °C overnight. DyLight®488 IgG (H + L) was used as the secondary antibody at 1/500 dilution for 1 h at room temperature. Images were acquired with a fluorescence microscope (Olympus, Japan) using DP Manager software.

### RNA extraction and qPCR analysis

Total RNA was extracted from lung homogenates (200 μL) using RNase, in accordance with the manufacturer’s protocol. qPCR was performed using One Step SYBR® Prime Script™ RT-PCR with QuantStudio 6 Flex Real-time PCR system (ABI, USA). The qPCR reaction schedule was as follows: 5 min at 42 °C and 10 s at 95 °C, followed by 40 cycles of 3 s at 95 °C, 30 s at 60 °C, and a melting curve step. The following gene-specific primers were used: Virus, 5′-GAC CGA TCC TGT CAC CTC TGA C-3′ and 5′-AGG GCA TTC TGG ACA AAG CGT CTA-3′; MOR, 5’- CCC AGT TCT TTA TGC GTT CCT-3’ and 5’-ATT AGC CGT GGA GGG GTG T-3’; DOR, 5’- ATG TAA AGA GGG CTG GGA ATG TAG-3’ and 5’- GGG TTG GTT TTG TTG TTT GGA-3’; GAPDH, 5′-ACC ACC ATG GAG AAG GCT GG-3′ and 5′-CTC AGT GTA GCC CAG GAT GC-3′. Gene expression was calculated using the 2^−ΔΔCT^ method.

### Cell isolation

The lymphocytes from the LN were gently disrupted with a sterile syringe plunger, filtered through a 90-μm nylon mesh, centrifuged, and resuspended in PBS containing 10% FBS. Lung homogenates were digested with collagenase (400 U/mL) and DNaseI, followed by incubation at 37 °C for 90 min, filtering through a 90-μm nylon mesh, and centrifugation at 1500 rpm for 5 min. The cells were then resuspended in 5 mL of RPMI 1640 medium containing 10% FBS and slowly added to a new 15 mL centrifuge tube containing 5 mL of a 25% Percoll density gradient. The lymphocytes from the lungs were isolated at the interface of the RPMI 1640 medium and Percoll density gradient after centrifugation at 2200 rpm for 20 min. Airway cells were removed from the lungs by three consecutive bronchoalveolar lavages using a cannula in 2 mL of 1 × PBS, and then centrifuged and resuspended. Lysis of RBCs in the lungs and BALF was performed when necessary. Concentrations of viable cells were determined using trypan blue exclusion.

### Cell stimulation

Aliquots of 10^6^ cells were seeded into 96-well plates. The effector memory CD8^+^ T cells: the cells were stimulated with presence or absence of influenza NP366-374 or PA224-233 peptides for 6 h at 37 °C and 5% CO_2_. Brefeldin A (BD Biosciences) was added at the first hour of incubation. These cells were harvested and washed with PBS/2% FBS for further staining.

### Flow cytometry analysis

Cells were incubated with FcγR-blocking mAb (CD16/32; BD Biosciences) for 15 min at 4 °C, washed, and stained with primary antibodies, such as anti-PerCP-CY5.5-CD8, anti-BV421-CD4, anti-FITC-CD44, or anti-APC-CD62L, for 30 min at 4 °C. For intracellular cytokine staining, cells were permeabilized in 100 μL of fixation/permeabilization solution for 20 min at 4 °C and washed with 1 mL of Perm/Wash Buffer. Cells were centrifuged and labeled with anti-PE-IFN-γ for 30 min at 4 °C, washed with 1 mL of Perm/Wash buffer, resuspended in 300 μL of Perm/Wash Buffer, and analyzed by flow cytometry.

### Statistical analyses

Results are presented as mean ± SD and were analyzed using GraphPad Prism 6.0 (GraphPad Software, USA). All data were analyzed by one-way ANOVA followed by Tukey’s post hoc test*.* A *p* value < 0.05 was considered statistically significant (^★^*p* < 0.05, ^★★^*p* < 0.01).

## Results

### MENK increased survival of mice infected with influenza virus

**Fig. 2:**
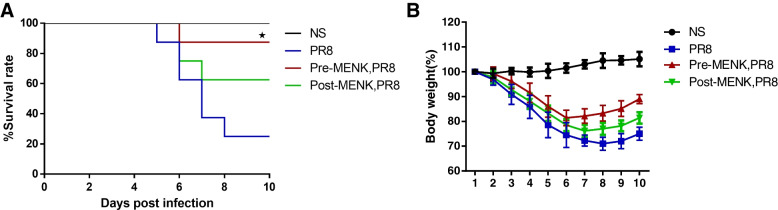
The influence of MENK on the mice infected with influenza virus. **A** the survival rate and **B** weight change. Each group of 8 mice were infected with 2 LD_50_ of influenza A virus (PR8), except for mice in the NS group. All the mice were monitored daily for 10 days. Data were analyzed using the log-rank(Mantel-Cox) test. ^★^*p *< 0.05 versus the PR8 group

The survival rate of mice in PR8 group was 25%, pre-MENK treatment significantly prolonged survival to 87.5% (*p* < 0.05 versus PR8 group). Similarly MENK treatment also improved the mice survival to 62.5% (Fig. 2A). The mice in the PR8 group showed a rapid weight loss from day 2 p.i., while mice intervened by MENK had relative weight loss. The pre-MENK group and post-MENK group showed stable weight gain on day 7 p.i. and day 8 p.i., respectively (Fig. 2B). The results demonstrated that pre-MENK effectively suppressed the lethal infection of influenza viruses, and prophylactic intervention of MENK was superior to therapeutic effect.

### MENK administration decreased viral replication

**Fig. 3:**
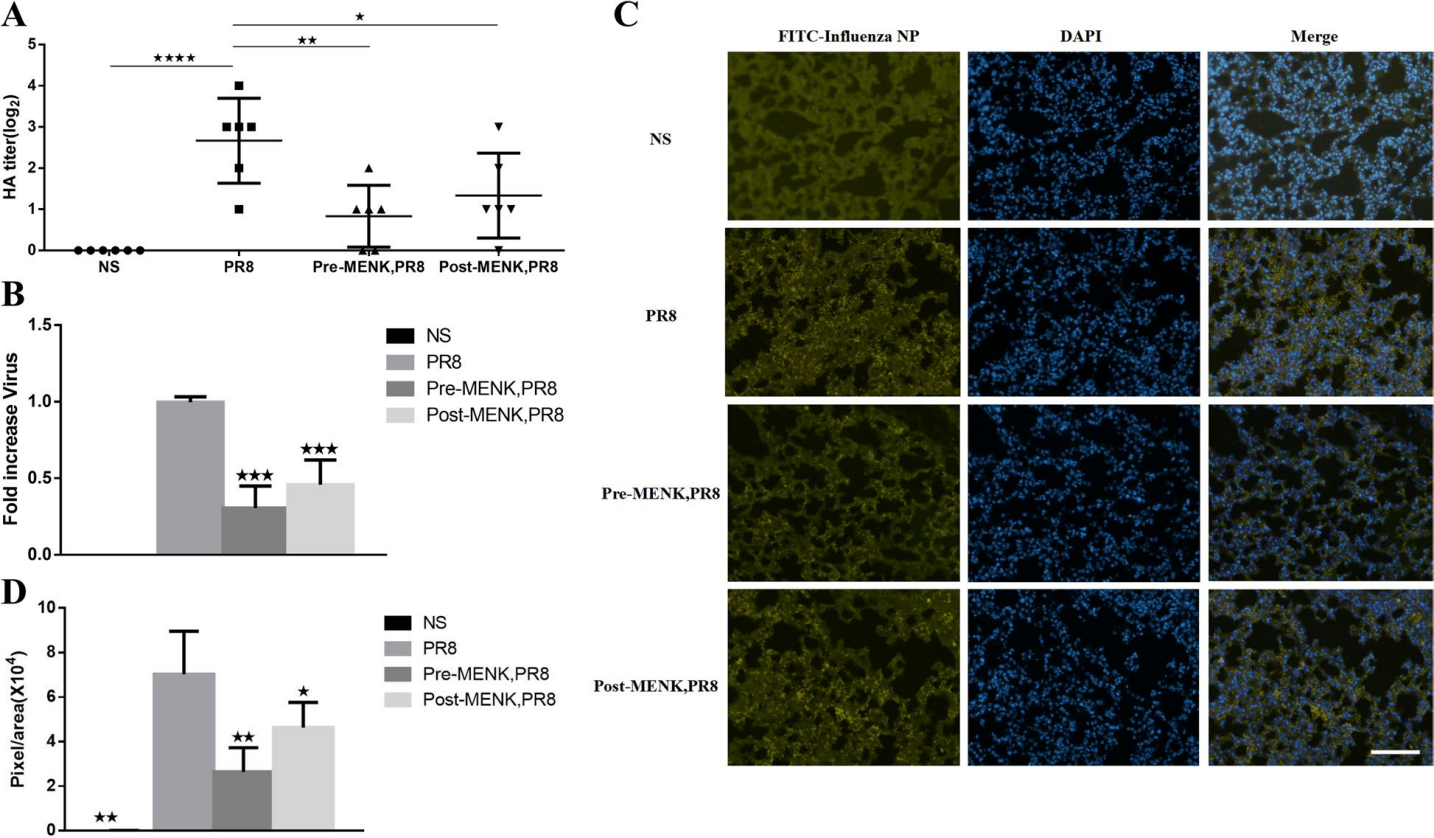
MENK administration inhibited virus replication in mice challenged with influenza virus. **A** HA titers and **B** Relative virus mRNA. The viral titers of supernatants of homogenized lung were determined by HA test and virus level in the lungs of mice was quantified by qPCR on day 4 p.i. **C** The localization and expression of influenza virus NP protein in lung tissue. **D** Panel represents quantified data of influenza virus NP protein expression. The data represent the mean ± SD of three independent experiments (each performed with *n* = 6 mice). Scale bar: 100 μm. ^★^*p* < 0.05, ^★★^*p* < 0.01, ^★★★^*p* < 0.001 versus the PR8 group

To evaluate the antiviral efficacy of MENK, we detected lung viral titers and mRNA in mice infected with influenza virus on day 4 p.i.. The lung viral titers were significantly higher in mice infected with PR8 virus, compared to the NS group (*p* < 0.001), the pre-MENK group (*p* < 0.01), and the post-MENK group (*p* < 0.05) (Fig. 3A). The data showed that the viral titres were reduced in mice treated with MENK compared to infected alone. Compared with the PR8 group, the relative viral levels was decreased to 0.31-fold in the pre-MENK group and 0.46-fold in the post-MENK group (*p* < 0.001) (Fig. 3B). The expression of influenza NP protein was localized by immunofluorescent staining, which showed it to be mainly expressed in the nuclei of virally infected cells. The fluorescence intensity of the lungs showed that the expression levels of influenza NP in mice from the pre-MENK group (2.5 × 10^4^ pixels/area) and post-MENK group (4.3 × 10^4^ pixels/area) were lower than that in the PR8-infected mice (7.0 × 10^4^ pixels/area) (*p* < 0.01) (Fig. 3C and D). These findings illustrated that both prophylactic and therapeutic treatments of MENK could effectively inhibit the replication of influenza virus.

### MENK administration upregulated opioid receptor

**Fig. 4:**
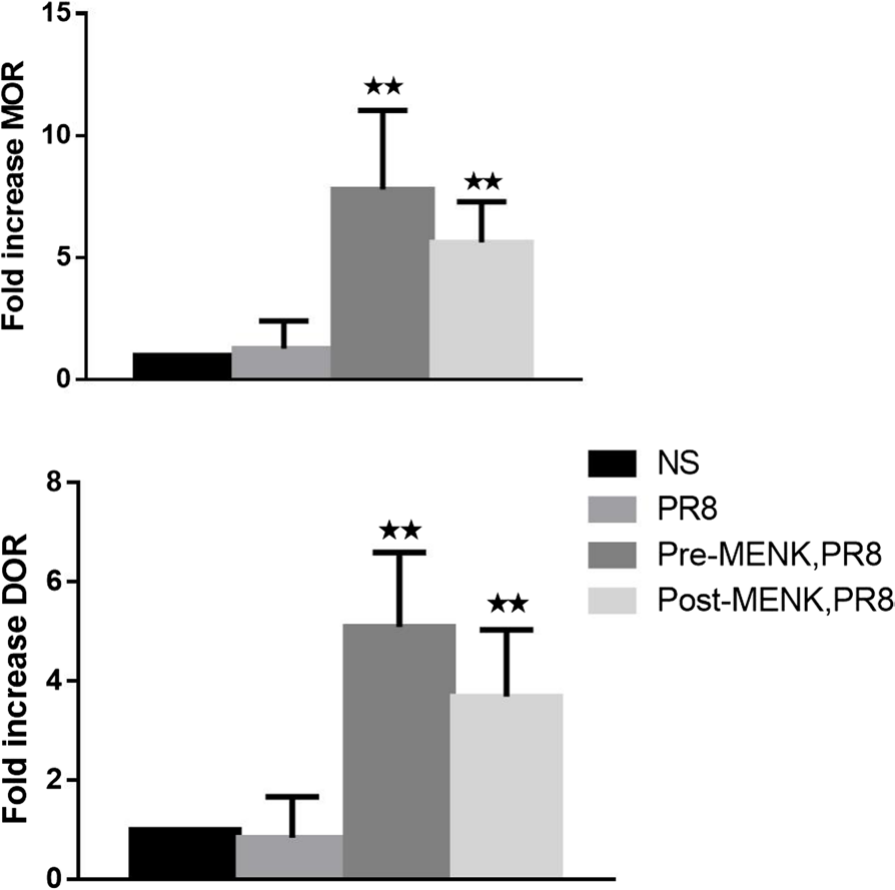
MENK treatment upregulated opioid receptors (MOR and DOR). Lungs were collected on day 10 p.i. Gene expression levels of opioid receptors (MOR and DOR) were quantified by qPCR. Results are presented as fold increase relative to the NS group. The data represent the mean ± SD of three independent experiments (each performed with *n* = 6 mice).^★^*p* < 0.05, ^★★^*p* < 0.01 versus the PR8 group

The MENK–MENKr (opioid receptor) axis maintains an equilibrium in cell replication and regulatory function, which is receptor-mediated, dose- and time-related, and reversible [16]. As shown in Fig. 4, there were no significant changes in expression of MOR and DOR in mice between the NS and PR8 groups on day 10 p.i.. However, there were marked increases in MOR and DOR in the pre-MENK group (MOR, 7.8-fold; DOR, 5.1-fold) and in the post-MENK group (MOR, 5.3-fold; DOR, 3.7-fold) compared with the levels in the PR8 group (*p* < 0.01). These results demonstrated that MENK upregulated the level of opioid receptors, which may be related to the mechanism of action of MENK administration against influenza.

### *MENK administration upregulated CD8*^+^*T cells*

**Fig. 5:**
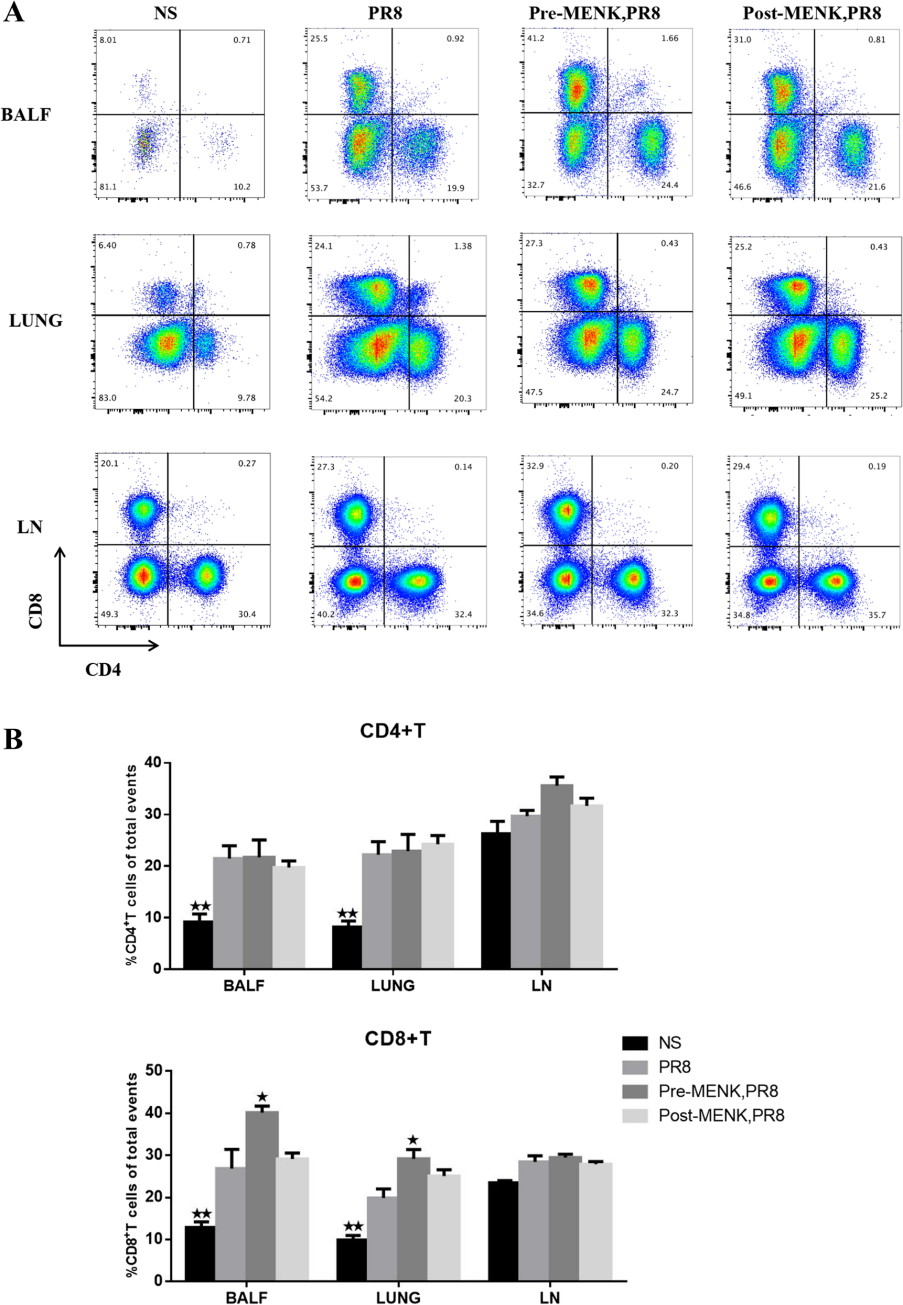
MENK increased the proportions of CD8^+^ T cells of mice infected with influenza virus. **A** Representative dot plots of CD4^+^ T and CD8^+^ T cells in BALF, lung, and LN of mice on day 10 p.i. **B** Panel presents the proportions of CD4^+^ T and CD8^+^ T cells. The data represent the mean ± SD of three independent experiments (each performed with *n* = 6 mice). ^★^*p* < 0.05, ^★★^*p* < 0.01 versus the PR8 group

Flow cytometry analysis showed that the proportions of CD4^+^ and CD8^+^ T cells in BALF and lungs of PR8-infected mice were significantly higher than in the NS group on day 10 p.i. (*p* < 0.01), while there was no significant change in LN (*p* > 0.05). Compared with the PR8 group, the proportions of CD8^+^ T cells in BALF and lungs of the pre-MENK group were significantly upregulated (*p* < 0.05), while CD4^+^ T cells also increased to some extent (*p* > 0.05). There was no statistical difference in the proportions of CD8^+^ T cells between post-MENK group and PR8 group (*p* > 0.05). The proportions of CD4^+^ and CD8^+^ T cells in LN had different degrees of upregulation with pre- and post-MENK treatment compared with those in PR8-infected mice, but the differences were not significant (*p* > 0.05) (Fig. 5A and B).

### *MENK administration upregulated CD4*^+^*/CD8*^+^*TM cells*

**Fig. 6:**
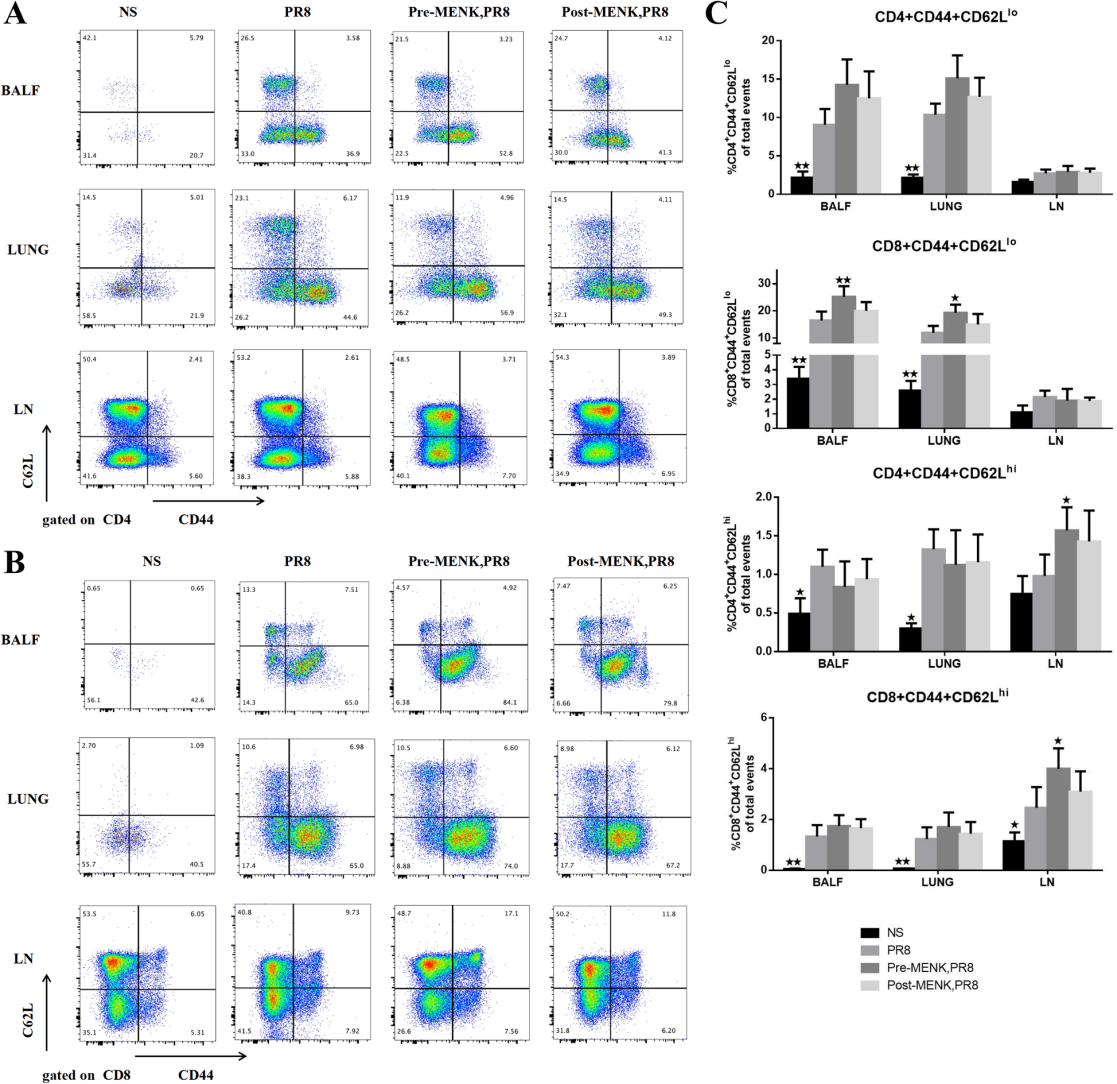
MENK upregulated CD8^+^ T_EM_ cells and CD4^+^/CD8^+^ T_CM_ cells of mice infected with influenza virus. **A** Representative dot plots of CD4^+^T_EM_ cells (CD4^+^CD44^hi^CD62L^lo^) and T_CM_ cells (CD4^+^CD44^hi^CD62L^hi^) in BALF, lung, and LN of mice on day 10 p.i. **B** Representative dot plots of CD8^+^ T_EM_ cells (CD8^+^CD44^hi^CD62L^lo^) and T_CM_ cells (CD8^+^CD44^hi^ CD62L^hi^) in BALF, lung, and LN of mice on day 10 p.i. **C** Panel presenting the proportions of CD4^+^/CD8^+^ T_EM_ cells and T_CM_ cells. The data represent the mean ± SD of three independent experiments (each performed with *n* = 6 mice). ^★^*p* < 0.05, ^★★^*p* < 0.01 versus the PR8 group

Memory T cells can be separated into two distinct subsets according to the respective expression levels of CD44 and CD62L, effector memory T cells (T_EM_, CD4^+^/CD8^+^ CD44^hi^CD62^lo^) and central memory T cells (T_CM,_ CD4^+^/CD8^+^CD44^hi^CD62^hi^) [26, 27]. As shown in Fig. 6A-C, the proportions of CD4^+^ T_EM_ and CD8^+^ T_EM_ cells in the BALF and lungs of PR8-infected mice markedly increased compared with those in uninfected mice on day 10 p.i. (*p* < 0.01). Pre-MENK administration enhanced the proportions of CD8^+^ T_EM_ cells in BALF and lungs to different degrees (*p* < 0.05), while post-MENK administration showed no such significant changes (*p* > 0.05). There were no significant differences in the proportions of CD4^+^ T_EM_ and CD8^+^ T_EM_ cells in LN of the four groups (*p* > 0.05). Influenza virus infection significantly increased the proportions of CD4^+^ T_CM_ and CD8^+^ T_CM_ cells in BALF and lungs (*p* < 0.01 or* p* < 0.05), and CD8^+^ T_CM_ cells in LN (*p* < 0.05) compared with the levels in the NS group. Compared with PR8-infected mice, pre-MENK administration upregulated the proportions of CD4^+^ T_CM_ and CD8^+^ T_CM_ cells in LN (*p* < 0.05), while having small effects on T_CM_ cells in BALF and lungs (*p* > 0.05). The proportions of CD8^+^ T_CM_ cells in the post-MENK group increased to different extents in BALF, lungs, and LN, but the amplitude of upregulation did not differ significantly from that in the PR8 group (*p* > 0.05). Our results suggest that pre-MENK administration upregulated both pulmonary and systemic CD4^+^/CD8^+^ T_M_ populations, which were beneficial for inducing rapid resistance to respiratory viruses and possibly enhancing immune protection against reinfection.

### *MENK administration upregulated effector CD8*^+^*TM cells*

**Fig. 7:**
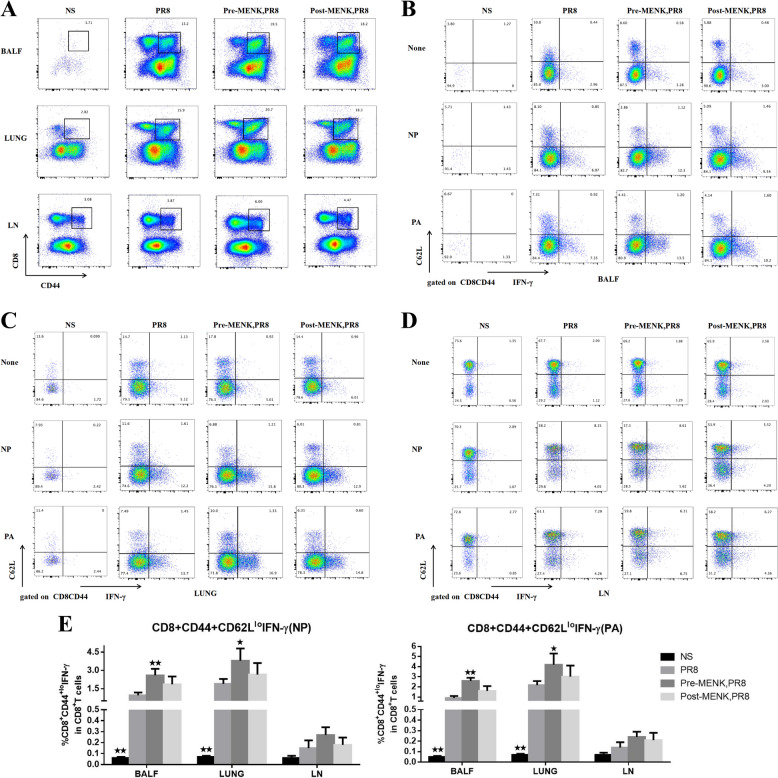
MENK upregulated effector memory CD8^+^ T cells of mice infected with influenza virus. **A** Representative dot plots of CD8^+^CD44^hi^ T cells in BALF, lung, and LN of mice on day 10 p.i.. Representative dot plots of CD62L^lo^IFN-γ^+^ cells in CD8^+^CD44^hi^ T cells in BALF (**B**), lung (**C**), and LN (**D**) of mice on day 10 p.i. Cells were stimulated with absence or presence of influenza NP366-374 or PA224-233 peptides and stained as described in Materials and Methods. **E** Panel presenting the proportion of NP/PA-effector memory CD8^+^ T cells (CD8^+^CD44^hi^CD62L^lo^ IFN-γ^+^) stimulated with NP or PA in BALF, lung, and LN. The data represent the mean ± SD of three independent experiments (each performed with *n* = 6 mice). ^★^*p* < 0.05, ^★★^*p* < 0.01 versus the PR8 group

The effector T_M_ cells were identified on the basis of IFN-γ production upon in vitro stimulation in the presence or absence of immunodominant epitopes of influenza NP366-374 or PA224-233 peptides. As shown in Fig. 7A-D, the proportions of NP/PA-effector CD8^+^ T_EM_ cells in BALF and lungs of the PR8 group were higher than those in the NS group (*p* < 0.01). Compared with PR8-infected mice, pre-MENK administration increased the proportions of NP/PA-effector CD8^+^ T_EM_ cells in BALF and lungs to different degrees (*p* < 0.01 or *p* < 0.05). Meanwhile, post-MENK administration upregulated those cells to a smaller extent (*p* > 0.05). In terms of NP/PA-effector CD8^+^ T_EM_ cells, the degrees of upregulation by pre- or post-MENK administration did not differ significantly in LN compared with the PR8 group (*p* > 0.05) (Fig. 7E). These data suggest that pre-MENK administration could give rise to a memory T-cell response against conserved epitopes NP and PA of influenza virus.

## Discussion

Recent studies have indicated that T-cell immunity is key to limiting the severity of disease caused by IAV infection, especially in cases where antibody immunization has been ineffective [28]. Incorporating IAV-specific cellular immunity into vaccine strategies provides an attractive approach for improving cross-reactive immunity against existing and new strains of influenza virus [29]. The administration of influenza vaccines via the respiratory tract had potential benefits over conventional parenteral administration, inducing immunity directly at the site of influenza exposure as well as reducing the discomfort caused by injection [30, 31]. Our team clarified that intranasally administered MENK significantly improved the survival rate, relieved acute lung injury, and decreased cytokine levels through suppressing TLR7–NF-κB p65 signaling pathways in mice infected with influenza virus [23]. Based on the confirmed innate immune regulation of MENK against IAV [24] and immunomodulatory effect on T cells [19–22], we hypothesized that MENK as new anti-IAV agent affects adaptive immune responses, such as upregulating T cells, and promoting the production of memory T cells, directed against virus-host interactions.

The present study demonstrated that MENK administered intranasally effectively decreased lung viral titers and inhibited viral replication, upregulated opioid receptor MOR and DOR, increased the proportions of CD8^+^ T cells, CD4^+^/CD8^+^ T_M_ cells and NP/PA-effector CD8^+^ T_CM_ to accelerate the clearance of influenza virus. The strategy of generating cellular immunity, especially strong memory T cell response in addition to antibodies, is an effective way to improve vaccination against influenza viruses [32, 33]. According to the anatomical distribution, phenotype, and function of memory T cells, they were divided into two major subsets, effector and central memory T cells [34]. The effector memory T cells (T_EM_) tend to localize in lung airways and induce a rapid effector status to virus infection, whereas central memory T cells (T_CM_) in secondary lymphoid tissues typically display the recall response to produce new effector cells that are subsequently recruited to lung airways mediated by the functions of chemokines [35, 36]. The experimental results demonstrated that MENK administration upregulated CD8^+^ T_EM_ in the airways and lungs, and CD4^+^/CD8^+^ T_CM_ in LN on day 10 p.i. The lung/airway-resident T_EM_ cells have rapid effector functions and directly exert cytolytic effects to control viruses. Meanwhile, T_CM_ cells proliferate and differentiate into effector memory cells in lymph nodes and are then recruited to the lungs to amplify the immune memory response. Our data suggested that MENK administration upregulated lung/airway-resident CD8^+^ T_EM_ to strengthen rapid effector functions, and increased CD4^+^/CD8^+^ T_CM_ in LN to promote the proliferation and differentiation into T_EM_ and then amplify the immune memory response. Our findings also implied that, in the early stage of influenza virus infection (on the 10th day of infection), the proportions of CD4^+^ T_M_ and CD8^+^ T_M_ cells in BALF, lungs, and LN were significantly higher than those of the traditional T_M_, which are derived from approximately 10% of effector T cells after viral clearance. These observations supported the concept of Kedzierska K [37] and Sckisel GD [38] that a stable T_CM_ and T_EM_ lineage was established in the early stage of influenza virus infection (the first week of infection).

Notably, MENK not only upregulated memory T cells, but also significantly increased the proportion of NP/PA-effector memory CD8^+^ T cells, which is crucial to induce cross-protective effects against influenza infection. IAV-specific memory T cells target a wide range of relatively conserved peptides derived from different influenza strains and subtypes. Although memory T cells play a more important role in heterologous immunity, this function is likely influenced by many factors including differences in host genetic background, types of and doses of priming or challenge virus, as well as the interval between priming and re-challenge. Our observations implied to some extent that MENK may be able to increase cross-protection against influenza infection by regulating effector memory cells that target conserved epitopes under appropriate influencing factors.

Adding T cell-targeted vaccine component that promotes effector memory CD8^+^ T cells for quickly recall antiviral CD8^+^ T effectors to the respiratory tract for controlling the early virus and/or inducing cross-protection is a new challenge for vaccines [39]. This supports the idea that MENK could be an immune modulator for anti-influenza. Integrating the results of our team, MENK administration has prophylactic and therapeutic effects on influenza virus infection [23, 24], and could upregulate T cells, memory T cells, and NP/PA-effector memory T cells. These findings indicated that MENK regulated the cellular immune response against primary influenza virus infection, and may be possible to enhance the cross-protection of memory response against reinfection under appropriate influencing factors. While the therapeutic administration of MENK (post-MENK) only adjusted the proportion of adaptive T cells to a certain extent, which did not reach significance. A possible reason for this was that MENK administration prior to virus infection might be able to induce the antiviral state in advance through upregulating the opioid receptors of MOR and DOR, and also implied that MENK was more suitable as an influenza prophylactic drug for wide application.

At present, the traditional drug oseltamivir that significantly decreased influenza-specific CD8^+^ effector T cells and tissue-resident and circulating effector memory and central memory CD8^+^ T cells [27], traditional vaccinations such as trivalent inactivated and attenuated vaccines are inadequate to provide immediate protection against sudden influenza outbreaks [40, 41]. In contrast, MENK, as an immunomodulatory polypeptide, could exert antiviral activity through upregulating T-cell-mediated adaptive immune responses against influenza virus. These characteristics are crucial to combat pandemic influenza virus strains. Collectively, our findings provide basic experimental data supporting the use of MENK as a more valuable immune modulator, offering new insights into developing novel antiviral drug and expanding the strategy aimed at preventing influenza pandemic. Further, we will have more work on the kinetics of CD8^+^ T cells’ response and whether these effects persist into the memory stage, label effector T and Tm cells based on their kinetics and response to specific peptide antigen. In this study, the increased CD4^+^ cells in LN could be Tfh type cells, which could enhance antibody production. Followed, we will explore the effect of MENK on antibody response, which may provide more favorable support for MENK to become immune modulator or prophylactic drug against influenza virus.

## Data Availability

The datasets in this study are available from the corresponding author on reasonable request.

## Abbreviations

BALF: Bronchoalveolar lavage fluid
CD62L: CD62 ligand
DOR: δ-Opioid receptor
hi: High
IAV: Influenza A virus
LN: Lymph nodes
lo: Low
MENK: Methionine encephalin
MOR: μ-Opioid receptor
NS: Normal saline
NP: Nucleoprotein
PR8: Influenza strains A/PR/8/34
PA: Polymerase acid
P.i.: Post-infection
qPCR: Quantitative PCR
T_CM_: Central memory T cell
T_EM_: Effector memory T cell
T_M_: Memory T cell
